# A Scoping Review of Artificial Intelligence Research in Rhinology

**DOI:** 10.1177/19458924231162437

**Published:** 2023-03-09

**Authors:** Gabriel Osie, Rhea Darbari Kaul, Raquel Alvarado, Gregory Katsoulotos, Janet Rimmer, Larry Kalish, Raewyn G. Campbell, Raymond Sacks, Richard J. Harvey

**Affiliations:** 1 Rhinology and Skull Base Research Group, Applied Medical Research Centre, 7800 University of New South Wales, Sydney, Australia; 2 School of Clinical Medicine, St Vincent's Healthcare Clinical Campus, Faculty of Medicine and Health, 7800 University of New South Wales, Sydney, Australia; 3 Woolcock Institute, University of Sydney, Sydney, Australia; 4 Faculty of Medicine, Notre Dame University, Sydney, Australia; 5 Department of Otolaryngology, Head and Neck Surgery, Concord General Hospital, University of Sydney, Sydney, Australia; 6 Faculty of Medicine, University of Sydney, Sydney, Australia; 7 Faculty of Medicine, Health and Human Sciences, Macquarie University, Sydney, Australia; 8 Department of Otolaryngology Head and Neck Surgery, 2205 Royal Prince Alfred Hospital, Sydney, Australia

**Keywords:** artificial intelligence, artificial neural network, convolutional neural network, machine learning, rhinology, phenotyping, endotyping, diagnostics, prognostics, sinus

## Abstract

**Background:**

A considerable volume of possible applications of artificial intelligence (AI) in the field of rhinology exists, and research in the area is rapidly evolving.

**Objective:**

This scoping review aims to provide a brief overview of all current literature on AI in the field of rhinology. Further, it aims to highlight gaps in the literature for future rhinology researchers.

**Methods:**

OVID MEDLINE (1946-2022) and EMBASE (1974-2022) were searched from January 1, 2017 until May 14, 2022 to identify all relevant articles. The Preferred Reporting Items for Systematic Reviews and Meta-analyses Extension for Scoping Reviews checklist was used to guide the review.

**Results:**

A total of 2420 results were identified of which 62 met the eligibility criteria. A further 17 articles were included through bibliography searching, for a total of 79 articles on AI in rhinology. Each year resulted in an increase in the number of publications, from 3 articles published in 2017 to 31 articles published in 2021. Articles were produced by authors from 22 countries with a relative majority coming from the USA (19%), China (19%), and South Korea (13%). Articles were placed into 1 of 5 categories: phenotyping/endotyping (n = 12), radiological diagnostics (n = 42), prognostication (n = 10), non-radiological diagnostics (n = 7), surgical assessment/planning (n = 8). Diagnostic or prognostic utility of the AI algorithms were rated as excellent (n = 29), very good (n = 25), good (n = 7), sufficient (n = 1), bad (n = 2), or was not reported/not applicable (n = 15).

**Conclusions:**

AI is experiencing an increasingly significant role in rhinology research. Articles are showing high rates of diagnostic accuracy and are being published at an almost exponential rate around the world. Utilizing AI in radiological diagnosis was the most published topic of research, however, AI in rhinology is still in its infancy and there are several topics yet to be thoroughly explored.

## Introduction

Artificial intelligence (AI) is an increasingly exciting area of research in medicine. Amongst other benefits, AI has the potential to automatically perform complex tasks with great speed^1^ and precision. Various applications of AI in medicine have already evolved from theoretical or proof-of-concept to being used in clinical practice, such as the automatic detection of atrial fibrillation via a smartphone or smartwatch-based ECG monitors^2,3^ or continuous glucose monitoring to prevent hypoglycaemia.^3,4^

**Figure 1.**
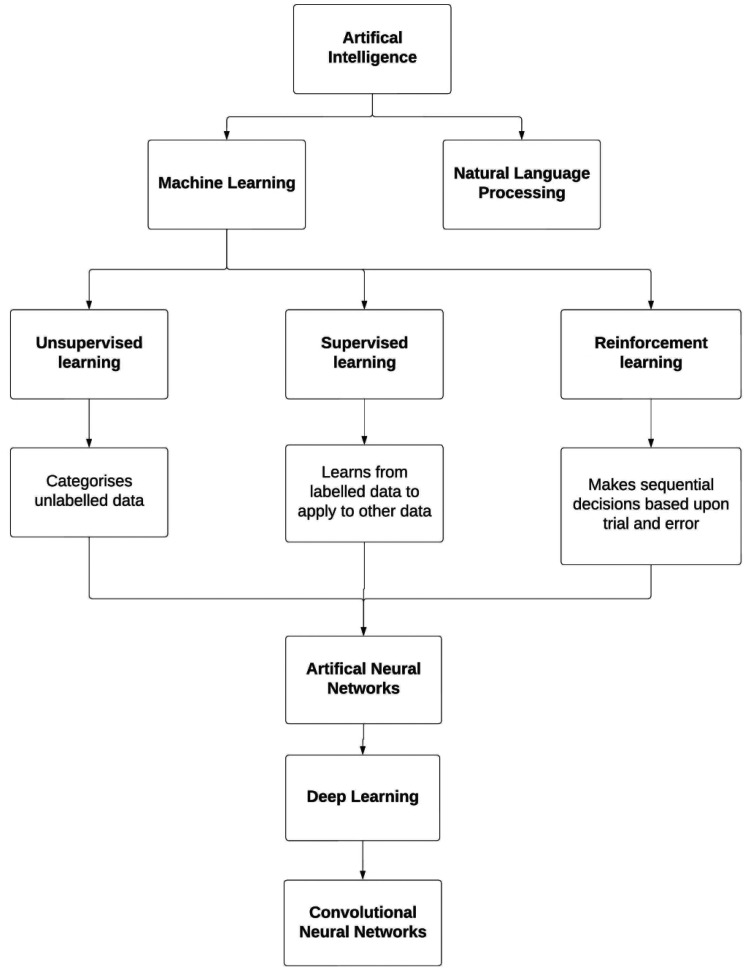
Summary of the types of artificial intelligence commonly used in medicine.

In this field there are several definitions that should be considered (Figure 1). Machine learning (ML) is a subset of AI that uses prior data to make improved decisions about future data.^5^ ML algorithms can be split into 3 main categories: supervised learning, unsupervised learning, and reinforcement learning.^6^ Supervised learning requires labelled datapoints for a ML algorithm to learn from, to later make predictions on unlabeled data.^5^ Unsupervised learning algorithms find patterns (eg, in cluster analysis) in unlabeled datasets.^5^ Reinforcement learning is the training of a ML model to make a sequence of decisions to solve a task through a process of trial and error.^7^ An artificial neural network (ANN) utilizes components of supervised and reinforcement learning to solve problems. ANNs use layers of processing to make sense of input information. The output of a layer becomes the input for the next layer, until it has been transformed into an output that can be used by the network.^8^ Deep learning is a subset of ANNs where at least 3 layers are used in the network.^9^ A convolutional neural network (CNN) is a type of ANN that processes data with grid-like structures (such as images).^10^ Natural language processing (NLP) is an entirely different subset of AI that allows computers to understand humans by converting language into data, that are processable by a computer.^11^

A considerable volume of research about possible applications of AI in the field of rhinology is available, yet no application has a widely used clinical application to date. This scoping review aimed to provide a brief overview of all current literature on AI in the field of rhinology. Further, it aimed to highlight implications for clinical practice and future research for rhinology researchers.

## Materials and Methods

Following a preliminary search of the literature on the usages of AI in rhinology, a scoping review was conducted. The Preferred Reporting Items for Systematic Reviews and Meta-analyses Extension for Scoping Reviews^12^ (PRISMA-ScR) checklist was used to guide the review. Registration was not applied as PROSPERO does not accept scoping reviews, literature reviews, or mapping reviews.

### Eligibility Criteria

Eligible studies described an application of AI to solve a clinical question in the field of rhinology. Articles needed to be published since 2017 to capture the most up-to-date literature in this rapidly expanding field of research.^13^ Unpublished literature is often reported in scoping reviews,^12^ as one of its purposes is to map a body of knowledge to identify gaps in research.^14^ Hence conference and poster abstracts were eligible for inclusion provided they had been presented since 2020. Grey literature from prior to 2020 were excluded. Articles were excluded if AI was only used in the statistical analysis of a paper, or if the use of AI was only to calculate radiation dosages in oncological settings. Articles that described the automatic segmentation of nasopharyngeal cancers in radiological scans were excluded if they were utilized for the purpose of determining radiation therapy/treatment. Works without original data, animal studies, and studies unavailable in English were also excluded.

### Information Sources

A systematic electronic search was performed for relevant studies using the Ovid MEDLINE (1946-2021) and EMBASE (1974-2021) databases from January 1, 2017 until the May 14, 2022 using a defined search strategy (Table 1). AI terms including “DEEP LEARNING” and “articifial adj2 intelligen*,” rhinology terms including “NOSE” and “Rhin*,” and investigation terms including “Endosco*” and “MRI*” were combined using the Boolean operators (AND and OR) to broaden and limit the search where appropriate. A manual bibliographic screen from the included and other relevant studies was performed to search for additional relevant articles undetected by the original search. An attempt was made to contact authors of studies where the full-text was not found.

**Table 1.** Search Strategy. Table of Terms Used in MEDLINE (2017-2022) and EMBASE (2017-2022) Databases. Search Performed on May 14, 2022.

Artificial intelligence terms	Rhinology terms	Investigation terms
1. AI 2. artificial adj2 intelligen* 3. Automat* 4. Convolutional 5. DEEP LEARNING 6. Deep learn* 7. MACHINE LEARNING 8. Machine adj2 learn* 9. MobileNet 10. Neural network 11. Pseudo-label* 12. ResNet 13. Self-train* 14. Self-taught* 15. Semi-supervised 16. SENet 17. Supervised 18. Unsupervised	19. Adenoid* 20. Ethmoid* 21. Frontal* adj2 sinus* 22. Inverted adj2 pap* 23. Maxil* 24. Meatal* 25. Meatus* 26. Nasal* 27. Naso* 28. NOSE 29. Nose* 30. Olfact* 31. Rhin* 32. Septal* 33. Septum* 34. Sinonas* 35. Sinus* 36. Smell* 37. Spheno* 38. AERD 39. AFRS 40. Allerg* 41. Atop* 42. CCAD 43. Central compartment 44. CRS* 45. eCRS* 46. fung*	47. CT 48. Comput* 49. Endonasal* 50. Endosco* 51. Fib??optic* 52. Intranasal* 53. Magnet* 54. MRI* 55. Exam* adj3 (nasal* OR nose*) 56. Nasendo* 57. Nasopharyngolaryngosc* 58. Nasopharyngosc* 59. Rhinoscop* 60. PET 61. Positron* 62. Scop* 63. Tomograph*
64. 1-18 OR 65. 19-46 OR 66. 47-63 OR 67. 64 AND 65 AND 66 68. 67 Limit to English and human 69. 68 Limit 2017-current		

### Study Selection

Duplicate studies were automatically removed using the OVID duplicate removal function, and later, manually. The remaining studies were exported to Rayyan (Qatar Computing Research Institute, Qatar),^15^ an online review tool, for screening against the eligibility criteria outlined above. Study selection was performed by 2 authors (GO and RK); uncertainties were resolved by consensus. Studies were screened in 3 phases: first by title, then by abstract, and finally by full-text. Articles that met the eligibility criteria were included for data collection.

### Data Collection

Data extracted from individual studies were recorded in Microsoft Excel for Mac (Version 16.63.1).^16^ Data fields collected included names of first and second authors, year of publication, study design, number of participants in total, as well as training, validation, and testing sets, demographic data of participants, country of publication, journal of publication, field of research, anatomical structures defined (if any), pathologies identified (if any), type of AI used, specific AI programs, algorithms, and software used, and diagnostic or prognostic accuracy (if applicable). There were several possible metrics of diagnostic accuracy. In order of preference, receiver operator characteristics area under the curve (ROC-AUC), Dice coefficient, and accuracy were used, however other metrics were satisfactory. Diagnostic or prognostic accuracy was rated as excellent if the ROC-AUC (or next best metric) was more than 0.9, very good if 0.8-0.9, good if 0.7-0.8, sufficient if 0.6-0.7, or bad if 0.5-0.6.

### Critical Appraisal of Individual Sources of Evidence

A critical appraisal or risk of bias assessment of individual studies is not typically performed in scoping reviews as their purpose is to identify the available evidence on a topic regardless of the methodological quality.^12^

## Results

### Study Selection

**Figure 2.**
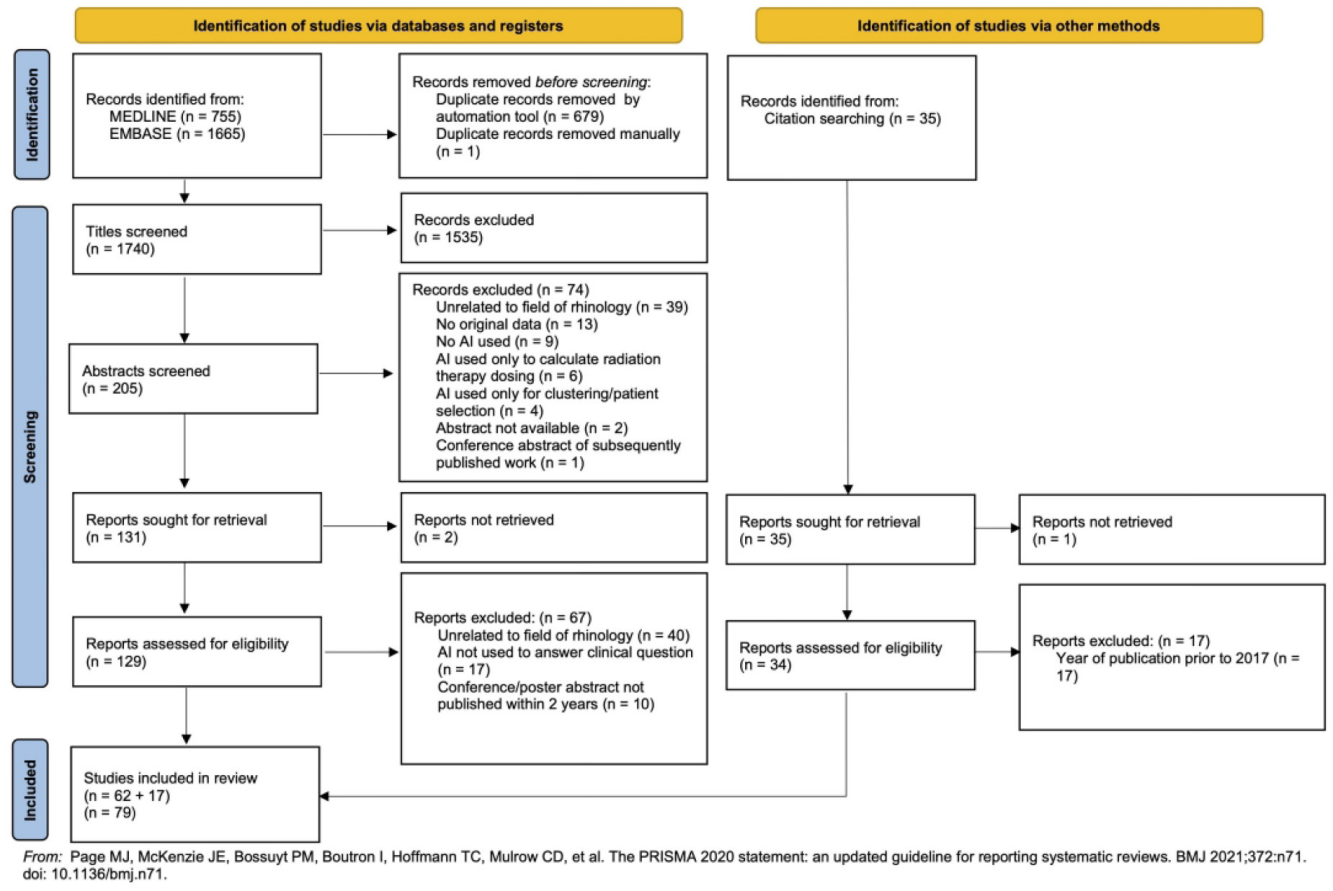
Preferred reporting items for systematic reviews and meta-analyses extension for scoping reviews flow chart highlighting the study selection process.

The search strategy yielded a total of n = 2420 studies. After the removal of 680 duplicates, n = 1740 titles were screened. Abstract screening was performed on 205 articles. Full-text analysis was performed on 129 articles (2 full-texts were unable to be located), resulting in 62 articles being included from the search. An additional 35 records were identified and screened from manual citation searching, of which 17 were included in the final analysis (7 of which were from journals not indexed by MEDLINE or EMBASE), leading to a combined total of 79 articles (Figure 2).

### Characteristics

**Figure 3.**
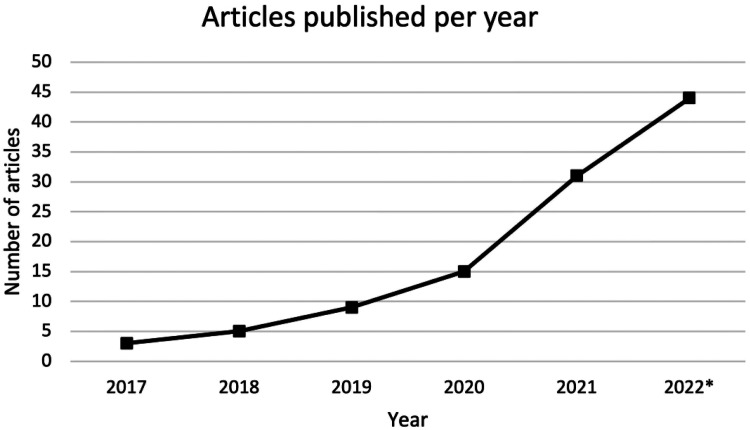
Number of articles on artificial intelligence in the field of rhinology since 2017.

Of the 79 articles identified, 3 were published in 2017,^17–19^ 5 were published in 2018,^20–24^ 9 were published in 2019,^25–33^ 15 were published in 2020,^34–48^ 31 were published in 2021,^49–79^ and 16 were published in 2022^80–95^ until May 14th (Figure 3). Study designs of the included articles were either cross-sectional (n = 61)^19,21,22,24,25,29,31–35,37–48,50–52,54–56,58,59,62–65,67,69,71–82,84–95^ or cohort studies (n = 18).^17,18,20,23,26–28,30,36,49,53,57,60,61,66,68,70,83^ Of the cross-sectional studies, 7 were presented at scientific conferences as abstracts,^20,32,40,47,56,78,95^ and 1 was presented at a conference as a poster.^77^

*16 articles were published until May 14, 2022. Prorated for a full year, this approximates to 44 articles.

Articles were published in journals of otolaryngology (n = 19),^17,18,23,26,28,30,36,38,39,42,46,52,57,65,72,77,86,87,94^ radiology (n = 21),^19,22,25,29,31,33,34,45,48,51,54,55,58,67,73,74,81,83–85,95^ medical sciences (n = 19),^32,41,44,47,53,61,63,66,68–70,76,78–80,89–91,93^ or other areas of medicine (n = 20).^20,21,24,27,35,37,40,43,49,50,56,59,60,62,64,71,75,82,88,92^ Countries of publication included United States of America (n = 15),^18,19,23,26,30,33,35,36,39,46,65,68,72,86,93^ China (n = 15),^24,37,40,45,49,67,69,74–76,80,84,87,92,94^ South Korea (n = 10),^28,29,52,54,55,57,64,82,85,89^ Germany (n = 8),^25,34,50,51,60,61,77,91^ Japan (n = 5),^27,31,58,62,63^ Hong Kong (n = 3),^48,73,81^ Indonesia (n = 3),^32,47,79^ Taiwan (n = 3),^41,53,83^ Australia (n = 2),^38,42^ Italy (n = 2),^22,59^ Netherlands (n = 2),^66,78^ and Brazil,^21^ Finland,^88^ France,^20^ Morocco,^17^ Poland,^43^ Russia,^44^ Singapore,^70^ Switzerland,^71^ Turkey,^95^ United Kingdom,^56^ and Vietnam^90^ (each n = 1).

**Figure 4.**
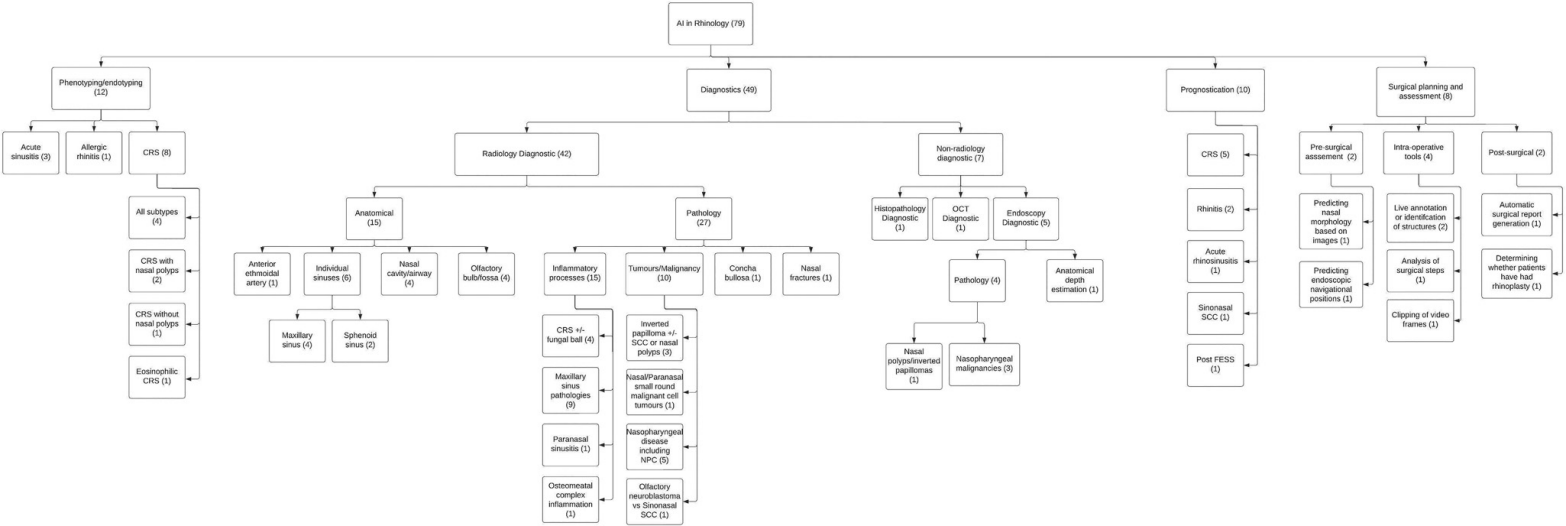
Major applications of artificial intelligence in rhinology flow chart.

Applications of AI in rhinology were arranged into 5 major categories (Figure 4):

1. *Phenotyping or endotyping*

There were 12 articles that used AI for phenotyping or endotyping pathology.^17,18,20,23,28,32,47,65,72,76,79,87^ The articles used either cluster analysis (n = 7)^17,18,20,23,28,65,79^ or ML (n = 5)^32,47,72,76,87^ in pathologies such as acute sinusitis (n = 3),^32,47,79^ allergic rhinitis (AR) (n = 1),^20^ and chronic rhinosinusitis (CRS) (n = 8).^17,18,23,28,65,72,76,87^ Diagnostic utility was rated as excellent (n = 5)^32,47,72,76,79^ or very good (n = 1),^87^ or was not applicable/not reported (n = 6)^17,18,20,23,28,65^ (Appendix 1).

2. *Radiology Diagnostics*

There were 42 articles that used an application of AI for radiology/imaging diagnostics in rhinology.^19,21,22,25,26,29,31,38–43,45,46,48–51,54,55,58,59,62–64,67,69,75,78,80–86,89,91–93,95^ Imaging tools used include CT (n = 25),^21,22,25,26,38–43,45,46,55,58,59,63,69,75,81–83,89,91,92,95^ MRI (n = 10),^19,48–51,78,80,84,86,93^ and X-ray (n = 6),^29,31,54,62,64,85^ with 1 article using both CT and MRI.^67^ ML techniques including CNNs and deep learning were utilized by all 42 studies.

Of the 42 articles total, 15 aimed to identify or characterize anatomical structures^21,22,25,38,41,43,45,50,51,59,78,91–93,95^ including the location of the anterior ethmoid artery (n = 1),^38^ the maxillary sinus (n = 4),^21,41,43,45^ the inferior turbinate (n = 1),^41^ the nasal cavity (n = 4),^25,59,91,93^ the olfactory bulb (n = 3),^50,51,78^ the depth of the olfactory fossa (n = 1),^95^ the sphenoid sinus (n = 2),^22,92^ and the entire upper airway (n = 1).^93^ Diagnostic accuracy was rated as excellent (n = 5),^21,45,59,91,92^ very good (n = 5),^38,41,51,93,95^ good (n = 1),^50^ or was not applicable/not reported (n = 4)^22,25,43,78^ (see Appendix 2).

The remaining 27 articles aimed to identify or differentiate between various pathologies^19,26,29,31,39,40,42,46,48,49,54,55,58,62–64,67,69,75,80–86,89^ including concha bullosa (n = 1),^42^ CRS (n = 3),^39,46,83^ fungal ball (n = 1),^82^ inverted papilloma (n = 3),^19,69,86^ nasal cancers (n = 4),^19,49,63,86^ maxillary defects (n = 1),^75^ maxillary sinus lesions (n = 3),^55,58,81^ maxillary sinusitis (n = 6),^29,31,54,62,64,85^ nasal fractures (n = 1),^89^ nasopharyngeal cancers (n = 4)^40,48,67,80^ or other pathologies (n = 1),^84^ and osteomeatal complex inflammation (n = 1).^26^ Diagnostic accuracy was rated as excellent (n = 12),^39,40,42,46,48,49,67,69,80,82,84,89^ very good (n = 12),^19,26,29,31,54,58,62–64,81,83,86^ or good (n = 3)^55,75,85^ (see Appendix 3).

3. *Prognostication*

There were 10 articles that used AI to predict prognosis or outcomes in rhinological issues.^27,30,36,53,57,60,61,66,68,88^ Aims of studies included prediction of outcomes in CRS (n = 3),^57,60,88^ AR (n = 1),^53^ sinonasal SCC (n = 1),^27^ and acute rhinosinusitis (n = 1),^66^ olfactory loss in rhinitis (n = 1)^61^ and CRS (n = 2),^30,68^ and predicting SNOT scores after functional endoscopic sinus surgery (n = 1).^36^ The articles used AI with ML (n = 8)^27,36,53,57,61,66,68,88^ or cluster analysis (n = 2).^30,60^ Prognostic accuracy was rated as excellent (n = 1),^27^ very good (n = 3),^53,57,60^ good (n = 2),^68,88^ sufficient (n = 1),^61^ bad (n = 1),^66^ or was not applicable/not reported (n = 2)^30,36^ (Appendix 4).

4. *Non-radiological diagnostics*

There were 7 articles that discussed AI applications for non-radiological diagnostic tools^24,33,37,44,52,70,94^ such as endoscopy (n = 5),^24,33,52,70,94^ histopathology (n = 1),^37^ and optic coherence tomography (OCT) (n = 1).^44^ All 7 papers utilized CNNs. The aims of the papers ranged from detecting nasopharyngeal carcinomas (n = 4),^24,37,70,94^ differentiating nasal polyps from inverted papillomas (n = 1),^52^ automatically identifying anatomical structures (n = 1)^33^ to differentiating chronic rhinitis (n = 1).^44^ ML techniques were utilized in all 7 studies. Diagnostic accuracy was rated as excellent (n = 3),^37,44,94^ very good (n = 2),^52,70^ good (n = 1),^24^ or was not applicable/not reported (n = 1)^33^ (see Appendix 5).

5. *Surgical planning and assessment*

The use of AI in surgical planning and assessment was explored by 8 studies.^34,35,56,71,73,74,77,90^ Each study was unique in their objective, including prediction of nasal bone morphology based on photographs of the nose (n = 1),^90^ automated analysis of surgical steps and identification of structures during pituitary surgery (n = 2),^56,71^ real-time 3-dimentional anatomical annotation during transnasal surgery (n = 1),^73^ automating the generation of operation reports in sinus surgery (n = 1),^77^ prediction of navigational positions during endoscopy (n = 1),^34^ automated prediction of whether patients had had rhinoplasty based on mobile photographs (n = 1),^35^ and automated clipping of nasal endoscopic videos (n = 1).^74^ ML was utilized in 7 of the studies^34,35,56,71,73,74,90^; NLP was used in 2 of the studies.^34,77^ Accuracy was rated as excellent (n = 3),^56,74,90^ very good (n = 2),^35,73^ bad (n = 1),^34^ or was not applicable/not reported (n = 2)^71,77^ (see Appendix 6).

## Discussion

The majority of publications on the use of AI in rhinology were in diagnostics (n = 49); most of these articles (n = 42) discussed radiological diagnostics in the form of CT, MRI, or X-ray imaging to automate identifying anatomical and pathological findings with CNNs, while the remaining (n = 7) detailed other diagnostic tools such as nasal endoscopy, OCT, or histopathology. Phenotyping/Endotyping was the next most common category of AI usage in rhinology (n = 12). The majority of these articles utilized cluster analysis to generate novel sub-type groups in pathologies such as CRS, acute sinusitis, and AR. In the prognostication group (n = 10), ML techniques (unsupervised or semi-supervised) and cluster analysis were used to predict outcomes or responses to treatments most commonly in CRS and AR. Surgical planning and assessment in rhinology was the least common area of AI research (n = 8), with newly evolving CNNs to aid pre-surgical assessment of nasal morphology and endoscopic navigational positions, intra-operative assessment with live annotation, analysis of surgical steps and video clipping, and post-operative tools such as surgical report generation and determination of prior rhinoplasty. The higher volume of AI rhinology research in radiological diagnostics compared to topics such as surgical planning and assessment is perhaps explained by a larger foundation of AI research in radiology in medicine as a whole, which is probably magnified by the investments in the field by tech giants such as Google.^96,97^ This would allow researchers to broadly apply previously published AI techniques to their own rhinological niche, whereas developing an AI for surgical planning and assessment requires a more novel technological approach. Throughout the articles, various AI programs were used; notably U-Net, a CNN designed for biomedical image segmentation and Res-Net, an ANN deigned for image recognition were most common.

Articles frequently reported high diagnostic accuracy of their AI algorithms, with 36.7% having an ROC-AUC (or other similar metric) rated as excellent, 31.6% rated as very good, 8.9% as good, 1.3% as sufficient, 2.5% as bad, and 19.0% not reporting.

### Implications for Clinical Practice

There are several promising areas in the field where AI has the potential to augment a rhinologist's practice. Although the risk of being entirely replaced by AI and robots is slim,^98^ there are potential ethical and legal issues^99^ with the implementation of AI in medicine which need to be addressed before AI can be considered for adoption into mainstream clinical practice.

In supervised learning models, data need to be labelled to train the ML algorithms. If the data is labelled incorrectly, the algorithm learns incorrectly, amplifying bias. This idea of ‘garbage in, garbage out’^100^ has been prevalent in computing since prior to the development of AI,^101^ and in the field of AI is commonly referred to as algorithm bias.^102^ A meta-analysis comparing the decisions of a ML software, called Watson For Oncology, to a multidisciplinary team of experts, found discordance in the decision making for treatment choices in lung cancers.^103^ This was partially explained by the use of synthetic data to train the algorithm^104^ as well as patient demographic differences.^103^ Very large amount of precisely labelled real data is essential for the development of supervised ML algorithms.^5,105^ It is also important that the data used to train ML algorithms is reflective of the diversity of the population. A landmark study in 2018 demonstrated that facial recognition AI developed by companies including IBM and Microsoft performed significantly worse at recognizing darker-skinned females than lighter-skinned males, with error rates up to 34.7% compared to 0.8%, respectively.^106^ The algorithmic bias was attributed to the databases used to train the AI being disproportionately comprised of lighter-skinned subjects.^106,107^

Reinforcement learning models such as ANNs, and unsupervised leaning models such as cluster analyses, often use ‘black box’ algorithms, which are AI systems in which the methodology for obtaining an output is hidden to the operator.^108^ An argument can be made in support of block box algorithms, as theoretically it shouldn’t matter how an algorithm gets to an answer if that the answer is always correct.^109^ The counterargument is that these algorithms don’t always produce a correct answer and that transparency is essential in bias detection.^110^ Furthermore, clinicians are also more likely to utilize AI if they are able to make sense of how the AI makes its decisions.^111^

Another challenge for implementing AI in medicine is navigating liability regimens. At present, clinicians would be liable if they acted against best practice due to the advice of an AI algorithm.^99,112^ In the future, if (or when) AI becomes more routine, we may have the corresponding case where clinicians could be liable if they act against the advice of the AI.

### Implications for Future Research

The obvious gaps in the research identified from this review are the comparative lack of publications about AI in phenotyping or endotyping pathologies, prognostication, non-radiological diagnostics, and surgical planning and assessment in comparison to radiological diagnostics.

However, the most important gap is the shortage of large datasets which are needed to develop rigorous algorithms. As previously discussed, large datasets of precisely labelled data are needed for supervised learning models to prevent algorithm bias. Before widespread adoption of AI into rhinology clinical practice, there needs to be a collaborative effort to collect appropriate datasets that reflect the demographics of the population. National registries have been used in orthopaedics^113^ and cardiology^114^ for this purpose. However, with the increased volume of patients in national registries may come a decrease in the quality of the data.^115^

### Conclusions

AI research is increasingly common, with publications per year from around the globe increasing at an almost exponential rate. Utilizing AI in radiological diagnosis was the most published topic of research, however, AI in rhinology is still in its infancy and there are several topics in prognostication and surgical planning yet to be thoroughly explored.

## Supplemental Material

sj-docx-1-ajr-10.1177_19458924231162437 - Supplemental material for A Scoping Review of Artificial Intelligence Research in Rhinology Click here for additional data file. Supplemental material, sj-docx-1-ajr-10.1177_19458924231162437 for A Scoping Review of Artificial Intelligence Research in Rhinology by Gabriel Osie, Rhea Darbari Kaul, Raquel Alvarado, Gregory Katsoulotos, Janet Rimmer, Larry Kalish, Raewyn G. Campbell, Raymond Sacks and Richard J. Harvey in American Journal of Rhinology & Allergy

sj-docx-2-ajr-10.1177_19458924231162437 - Supplemental material for A Scoping Review of Artificial Intelligence Research in Rhinology Click here for additional data file. Supplemental material, sj-docx-2-ajr-10.1177_19458924231162437 for A Scoping Review of Artificial Intelligence Research in Rhinology by Gabriel Osie, Rhea Darbari Kaul, Raquel Alvarado, Gregory Katsoulotos, Janet Rimmer, Larry Kalish, Raewyn G. Campbell, Raymond Sacks and Richard J. Harvey in American Journal of Rhinology & Allergy

sj-docx-3-ajr-10.1177_19458924231162437 - Supplemental material for A Scoping Review of Artificial Intelligence Research in Rhinology Click here for additional data file. Supplemental material, sj-docx-3-ajr-10.1177_19458924231162437 for A Scoping Review of Artificial Intelligence Research in Rhinology by Gabriel Osie, Rhea Darbari Kaul, Raquel Alvarado, Gregory Katsoulotos, Janet Rimmer, Larry Kalish, Raewyn G. Campbell, Raymond Sacks and Richard J. Harvey in American Journal of Rhinology & Allergy

sj-docx-4-ajr-10.1177_19458924231162437 - Supplemental material for A Scoping Review of Artificial Intelligence Research in Rhinology Click here for additional data file. Supplemental material, sj-docx-4-ajr-10.1177_19458924231162437 for A Scoping Review of Artificial Intelligence Research in Rhinology by Gabriel Osie, Rhea Darbari Kaul, Raquel Alvarado, Gregory Katsoulotos, Janet Rimmer, Larry Kalish, Raewyn G. Campbell, Raymond Sacks and Richard J. Harvey in American Journal of Rhinology & Allergy

sj-docx-5-ajr-10.1177_19458924231162437 - Supplemental material for A Scoping Review of Artificial Intelligence Research in Rhinology Click here for additional data file. Supplemental material, sj-docx-5-ajr-10.1177_19458924231162437 for A Scoping Review of Artificial Intelligence Research in Rhinology by Gabriel Osie, Rhea Darbari Kaul, Raquel Alvarado, Gregory Katsoulotos, Janet Rimmer, Larry Kalish, Raewyn G. Campbell, Raymond Sacks and Richard J. Harvey in American Journal of Rhinology & Allergy

sj-docx-6-ajr-10.1177_19458924231162437 - Supplemental material for A Scoping Review of Artificial Intelligence Research in Rhinology Click here for additional data file. Supplemental material, sj-docx-6-ajr-10.1177_19458924231162437 for A Scoping Review of Artificial Intelligence Research in Rhinology by Gabriel Osie, Rhea Darbari Kaul, Raquel Alvarado, Gregory Katsoulotos, Janet Rimmer, Larry Kalish, Raewyn G. Campbell, Raymond Sacks and Richard J. Harvey in American Journal of Rhinology & Allergy
