# Supplementary material for: A Scoping Review of Artificial Intelligence Research in Rhinology
Source: Am J Rhinol Allergy. 2023 Mar 9;37(4):438–48. doi: 10.1177/19458924231162437 (PMC10273866; doi:10.1177/19458924231162437)
Supplement: sj-docx-1-ajr-10.1177_19458924231162437 - Supplemental material for A Scoping Review of Artificial Intelligence Research in Rhinology [file sj-docx-1-ajr-10.1177_19458924231162437.docx]

| **Phenotyping or endotyping** | | | | | |
| --- | --- | --- | --- | --- | --- |
| **First author – Country of study** | **Year** | **Title** | **Pathology** | **Type of AI used** | **Diagnostic Utility** |
| Arfiani – Indonesia | 2019 | Kernel spherical K-means and support vector machine for acute sinusitis classification | Acute sinusitis | Cluster analysis | Excellent |
| Rustam – Indonesia | 2020 | Kernel perceptron algorithm for sinusitis classification | Acute sinusitis | Machine learning | Excellent |
| Rustam – Indonesia | 2021 | Kernel entropy based fuzzy C-means (KEFCM) for acute sinusitis | Acute sinusitis | Machine learning | Excellent |
| Caimmi – France | 2018 | Discriminating severe seasonal allergic rhinitis: results from a large nation-wide database | Allergic rhinitis | Cluster analysis | N/A |
| Liu – China | 2022 | Microvessel quantification by fully convolutional neural networks associated with type 2 inflammation in chronic rhinosinusitis | CRS | Convolutional neural network | Very good |
| Parsel – USA | 2021 | Differentiation of Clinical Patterns Associated With Rhinologic Disease | CRS | Unsupervised cluster analysis | N/A |
| Divekar – USA | 2017 | Unsupervised network mapping of commercially available immunoassay yields three distinct chronic rhinosinusitis endotypes | CRS | Unsupervised cluster analysis | N/A |
| Adnane – Morocco | 2017 | Using preoperative unsupervised cluster analysis of chronic rhinosinusitis to inform patient decision and endoscopic sinus surgery outcome | CRS | Unsupervised cluster analysis | N/A |
| Wu – China | 2021 | Artificial intelligence for cellular phenotyping diagnosis of nasal polyps by whole-slide imaging | CRS with nasal polyps | Semi-supervised convolutional neural network | Excellent |
| Kim – South Korea | 2019 | Unsupervised cluster analysis of chronic rhinosinusitis with nasal polyp using routinely available clinical markers and its implication in treatment outcomes | CRS with nasal polyps | Unsupervised cluster analysis | N/A |
| Lal – USA | 2018 | SNOT-22–based clusters in chronic rhinosinusitis without nasal polyposis exhibit distinct endotypic and prognostic differences | CRS without nasal polyps | Unsupervised cluster analysis | N/A |
| Thorwarth – USA | 2020 | Machine learning of biomarkers and clinical observation to predict eosinophilic chronic rhinosinusitis: a pilot study | eCRS | Artificial neural network | Excellent |
