# Supplementary material for: A Scoping Review of Artificial Intelligence Research in Rhinology
Source: Am J Rhinol Allergy. 2023 Mar 9;37(4):438–48. doi: 10.1177/19458924231162437 (PMC10273866; doi:10.1177/19458924231162437)
Supplement: sj-docx-2-ajr-10.1177_19458924231162437 - Supplemental material for A Scoping Review of Artificial Intelligence Research in Rhinology [file sj-docx-2-ajr-10.1177_19458924231162437.docx]

| **Radiology diagnostics – anatomical identification** | | | | | | |
| --- | --- | --- | --- | --- | --- | --- |
| **First author – Country of study** | **Year** | **Title** | **Imaging modality** | **Anatomical structure identified/defined** | **Type of AI used** | **Diagnostic utility** |
| Huang – Australia | 2020 | An artificial intelligence algorithm that differentiates anterior ethmoidal artery location on sinus computed tomography scans | CT | Anterior ethmoidal artery | Convolutional neural network | Very good |
| Pryzstanska – Poland | 2020 | Sexual dimorphism of maxillary sinuses in children and adolescents - A retrospective CT study. | CT | Maxillary sinus | Convolutional neural network | N/A |
| Xu – China | 2020 | Automatic CT image segmentation of maxillary sinus based on VGG network and improved V-Net. | CT | Maxillary sinus | Convolutional neural network | Excellent |
| Giacomini – Brazil | 2018 | Computed tomography-based volumetric tool for standardized measurement of the maxillary sinus | CT | Maxillary sinus | Convolutional neural network | Excellent |
| Kuo – Taiwan | 2020 | Application of intelligent automatic segmentation and 3D reconstruction of inferior turbinate and maxillary sinus from computed tomography and analyze the relationship between volume and nasal lesion | CT | Maxillary sinus, inferior turbinate | Back propagation neural network | Very good |
| Waldmann – Germany | 2022 | An effective simulation- and measurement-based workflow for enhanced diagnostics in rhinology | CT | Nasal cavity | Convolutional neural network | Excellent |
| Laura – Germany | 2019 | Automatic detection of the nasal cavities and paranasal sinuses using deep neural networks | CT | Nasal cavity and paranasal sinuses | Convolutional neural network | N/A |
| Desser – Germany | 2021 | Automatic segmentation of the olfactory bulb | MRI | Olfactory bulb | Convolutional neural network | Good |
| Estrada – Germany | 2021 | Automated olfactory bulb segmentation on high resolutional T2-weighted MRI | MRI | Olfactory bulb | Convolutional neural network | Very good |
| Postma – Netherlands | 2021 | Applying Olfactory Bulb Volume in the Clinic: Relating Clinical Outcome Measures to Olfactory Bulb Volume Using Convolutional Neural Networks | MRI | Olfactory bulb | Convolutional neural network | N/A |
| Dursun – Turkey | 2022 | Success of keros classification in paranasal sinus ct with deep learning method | CT | Olfactory fossa | Convolutional neural network | Very good |
| Leonardi – Italy | 2021 | Fully automatic segmentation of sinonasal cavity and pharyngeal airway based on convolutional neural networks. | CT | Sinonasal cavity and pharyngeal airway | Convolutional neural network, | Excellent |
| Wen – China | 2022 | Human identification performed with skull's sphenoid sinus based on deep learning | CT | Sphenoid sinus | Convolutional neural network | Excellent |
| Gibelli - Italy | 2018 | Volumetric assessment of sphenoid sinuses through segmentation on CT scan. | CT | Sphenoid sinus | Convolutional neural network | N/A |
| Xie – USA | 2022 | Automatic upper airway segmentation in static and dynamic MRI via anatomy-guided convolutional neural networks | MRI | Upper airway | Convolutional neural network | Very good |
