# Supplementary material for: A Scoping Review of Artificial Intelligence Research in Rhinology
Source: Am J Rhinol Allergy. 2023 Mar 9;37(4):438–48. doi: 10.1177/19458924231162437 (PMC10273866; doi:10.1177/19458924231162437)
Supplement: sj-docx-3-ajr-10.1177_19458924231162437 - Supplemental material for A Scoping Review of Artificial Intelligence Research in Rhinology [file sj-docx-3-ajr-10.1177_19458924231162437.docx]

| **Radiology diagnostics – detection of pathology** | | | | | | |
| --- | --- | --- | --- | --- | --- | --- |
| **First author – Country of study** | **Year** | **Title** | **Imaging modality** | **Pathology detected** | **Type of AI used** | **Diagnostic utility** |
| Parmar – Australia | 2020 | An artificial intelligence algorithm that identifies middle turbinate pneumatisation (concha bullosa) on sinus computed tomography scans | CT | Concha bullosa | Convolutional neural network | Excellent |
| Kuo – Taiwan | 2022 | Semi-Supervised Deep Learning Semantic Segmentation for3D Volumetric Computed Tomographic Scoring of Chronic Rhinosinusitis: Clinical Correlations and Comparison with Lund-Mackay Scoring | CT | CRS | Convolutional neural network | Very good |
| Humphries – USA | 2020 | Volumetric assessment of paranasal sinus opacification on computed tomography can be automated using a convolutional neural network | CT | CRS | Convolutional neural network | Excellent |
| Massey – USA | 2020 | Clinical validation of an Automated Deep-Learning-Based Algorithm for Quantitative sinus Computed Tomography Analysis | CT | CRS - Sinus opacification | Convolutional neural network | Excellent |
| Kim – South Korea | 2022 | Detection of maxillary sinus fungal ball via 3-D CNN-based artificial intelligence: Fully automated system and clinical validation | CT | CRS vs Fungal ball | Convolutional neural network | Excellent |
| Ren – China | 2021 | Deep learning framework for preoperative recognition of inverted papilloma and nasal polyp | CT | Inverted papilloma and nasal polyps | Convolutional neural network | Excellent |
| Liu – USA | 2022 | Deep learning classification of inverted papilloma malignant transformation using 3D convolutional neural networks and magnetic resonance imaging | MRI | Inverted papilloma and SCC | Convolutional neural network | Very good |
| Ramkumar – USA | 2017 | MRI-Based Texture Analysis to Differentiate Sinonasal Squamous Cell Carcinoma from Inverted Papilloma | MRI | Inverted papilloma and SCC | Semi-supervised machine learning | Very good |
| Ogawa – Japan | 2021 | Utility of CT texture analysis to differentiate olfactory neuroblastoma from sinonasal squamous cell carcinoma | CT | Olfactory neuroblastoma or Sinonasal SCC | Convolutional neural network | Very good |
| Chen – China | 2021 | Texture Analysis of Fat-Suppressed T2-Weighted Magnetic Resonance Imaging and Use of Machine Learning to Discriminate Nasal and Paranasal Sinus Small Round Malignant Cell Tumors | MRI | Nasal and paranasal small round malignant cell tumours | Deep learning | Excellent |
| Wang – China | 2021 | 3D morphometric quantification of maxillae and defects for patients with unilateral cleft palate via deep learning-based CBCT image auto-segmentation | CT | Maxillary defects | Convolutional neural network | Good |
| Hung – Hong Kong | 2022 | Automatic detection and segmentation of morphological changes of the maxillary sinus mucosa on cone beam computed tomography images using a three dimensional convolutional neural network | CT | Maxillary sinus lesions | Convolutional neural network | Very good |
| Jung – South Korea | 2021 | Deep Active Learning for Automatic Segmentation of Maxillary Sinus Lesions Using a Convolutional Neural Network | CT | Maxillary sinus lesions | Convolutional neural network | Good |
| Kuwana – Japan | 2021 | Performance of deep learning object detection technology in the detection and diagnosis of maxillary sinus lesions on panoramic radiographs, | CT | Maxillary sinus lesions | Deep learning | Very good |
| Lim – South Korea | 2022 | Aux-MVNet: Auxiliary classifier-based multi-view convolutional neural network for maxillary sinusitis diagnosis on paranasal sinuses view | X-Ray | Maxillary sinusitis | Convolutional neural network, | Good |
| Mori – Japan | 2021 | A deep transfer learning approach for the detection and diagnosis of maxillary sinusitis on panoramic radiographs | X-Ray | Maxillary sinusitis | Deep learning | Very good |
| Oh – South Korea | 2021 | Effective end-to-end deep learning process in medical imaging using independent task learning: application for diagnosis of maxillary sinusitis | X-Ray | Maxillary sinusitis | Deep learning | Very good |
| Kim – South Korea | 2019 | Deep Learning in Diagnosis of Maxillary Sinusitis Using Conventional Radiography. | X-Ray | Maxillary sinusitis | Convolutional neural network | Very good |
| Murata – Japan | 2019 | Deep-learning classification using convolutional neural network for evaluation of maxillary sinusitis on panoramic radiography | X-Ray | Maxillary sinusitis | Convolutional neural network | Very good |
| Jeon – South Korea | 2021 | Deep learning for diagnosis of paranasal sinusitis using multi-view radiograph | X-Ray | Maxillary sinusitis | Deep convolutional neural network | Very good |
| Seol – South Korea | 2022 | A study on 3D dep learning-based automatic diagnosis of nasal fractures | CT | Nasal fracture | Residual neural network | Excellent |
| Li – China | 2022 | Anatomical partition-based deep learning: an automatic nasopharyngeal MRI recognition scheme | MRI | Nasopharyngeal diseases | Deep learning convolutional neural network | Excellent |
| Deng – China | 2022 | The contrast-enhanced MRI can be substituted by unenhanced MRI in identifying and automatically segmenting primary nasopharyngeal carcinoma with the aid of deep learning models: An exploratory study in large-scale population of endemic area | MRI | Nasopharyngeal carcinoma | Densely connected convolutional network | Excellent |
| Qi – China | 2021 | Computer aided diagnosis and regional segmentation of nasopharyngeal carcinoma based on multimodality medical images | CT and MRI | Nasopharyngeal carcinoma | Convolutional neural network | Excellent |
| Ke – China | 2020 | Development of a self-constrained 3D DenseNet model in automatic detection and segmentation of nasopharyngeal carcinoma using magnetic resonance images | CT | Nasopharyngeal carcinoma | Convolutional neural network | Excellent |
| Wong – Hong Kong | 2020 | Convolutional neural network for discriminating nasopharyngeal carcinoma and benign hyperplasia on MRI | MRI | Nasopharyngeal carcinoma | Convolutional neural network | Excellent |
| Chowdhury – USA | 2019 | Automated classification of osteomeatal complex inflammation on computed tomography using convolutional neural networks. | CT | Osteomeatal complex inflammation | Convolutional neural network | Very good |
