# Supplementary material for: A Scoping Review of Artificial Intelligence Research in Rhinology
Source: Am J Rhinol Allergy. 2023 Mar 9;37(4):438–48. doi: 10.1177/19458924231162437 (PMC10273866; doi:10.1177/19458924231162437)
Supplement: sj-docx-4-ajr-10.1177_19458924231162437 - Supplemental material for A Scoping Review of Artificial Intelligence Research in Rhinology [file sj-docx-4-ajr-10.1177_19458924231162437.docx]

| **Prognostication** | | | | | |
| --- | --- | --- | --- | --- | --- |
| **First author – Country of study** | **Year** | **Title** | **Aim of AI** | **Type of AI used** | **Prognostic utility** |
| Nuutinen - Finland | 2022 | Using machine learning for the personalised prediction of revision endoscopic sinus surgery | Predicting need for revision FESS in patients with CRS | Machine learning | Good |
| Lotsch – Germany | 2021 | Machine-Learning Points at Endoscopic, Quality of Life, and Olfactory Parameters as Outcome Criteria for Endoscopic Paranasal Sinus Surgery in Chronic Rhinosinusitis | Outcomes for CRSwNP patients who underwent FESS | Semi-supervised cluster analysis | Very good |
| Kim – South Korea | 2021 | Subepithelial neutrophil infiltration as a predictor of the surgical outcome of chronic rhinosinusitis with nasal polyps | Predicting surgical outcomes in CRSwNP using subepithelial neutrophil infiltration | Machine learning | Very good |
| Huang – Taiwan | 2021 | Prenatal exposure to air pollutants and childhood atopic dermatitis and allergic rhinitis adopting machine learning approaches: 14-year follow-up birth cohort study. | Predicting allergic rhinitis based upon exposure to air pollutants | Machine learning | Very good |
| Fujima - Japan | 2019 | Machine-learning-based prediction of treatment outcomes using MR imaging–derived quantitative tumor information in patients with sinonasal squamous cell carcinomas: a preliminary study. | Prediction of treatment outcomes in sinonasal SCC | Machine learning | Excellent |
| Qi - Netherlands | 2021 | Explaining heterogeneity of individual treatment causal effects by subgroup discovery: an observational case study in antibiotics treatment of acute rhino-sinusitis | Predicting response to antibiotics in acute rhino-sinusitis | Machine learning | Bad |
| Lotsch – Germany | 2021 | Data Science-Based Analysis of Patient Subgroup Structures Suggest Effects of Rhinitis on All Chemosensory Perceptions in the Upper Airways | Identifying effects of rhinitis with olfactory loss on other chemosensory systems of upper airways | Machine learning | Sufficient |
| Ramakrishnan – USA | 2021 | Predicting olfactory loss in chronic rhinosinusitis using machine learning | Predicting olfactory loss in CRS patients | Machine learning | Good |
| Morse - USA | 2019 | Patterns of olfactory dysfunction in chronic rhinosinusitis identified by hierarchical cluster analysis and machine learning algorithms. | Predicting olfactory dysfunction in CRS patients using cytokines | Unsupervised cluster analysis | N/A |
| Chowdhury – USA | 2020 | Baseline mucus cytokines predict 22-item Sino-Nasal Outcome Test results after endoscopic sinus surgery | Predicting SNOT scores post FESS using cytokines | Machine learning | N/A |
