# Supplementary material for: A Scoping Review of Artificial Intelligence Research in Rhinology
Source: Am J Rhinol Allergy. 2023 Mar 9;37(4):438–48. doi: 10.1177/19458924231162437 (PMC10273866; doi:10.1177/19458924231162437)
Supplement: sj-docx-5-ajr-10.1177_19458924231162437 - Supplemental material for A Scoping Review of Artificial Intelligence Research in Rhinology [file sj-docx-5-ajr-10.1177_19458924231162437.docx]

| **Non-radiological diagnostics** | | | | | | |
| --- | --- | --- | --- | --- | --- | --- |
| **First author – Country of study** | **Year** | **Title** | **Diagnostic tool** | **Aim of AI diagnostics** | **Type of AI used** | **Diagnostic utility** |
| Xu - China | 2022 | Deep Learning for nasopharyngeal Carcinoma Identification Using Both White Light and Narrow-Band Imaging Endoscopy | Endoscopy | Detection of Nasopharyngeal Carcinoma | Deep convolutional neural network | Excellent |
| Diao – China | 2020 | Computer-Aided Pathologic Diagnosis of Nasopharyngeal Carcinoma Based on Deep Learning. | Histopathology diagnosis | Nasopharyngeal Carcinoma tissue diagnosis | Convolutional neural network | Excellent |
| Shu – Singapore | 2021 | Deep Learning-Guided Fibreoptic Raman Spectroscopy Enables Real-Time In Vivo Diagnosis and Assessment of Nasopharyngeal Carcinoma and Post-treatment Efficacy during Endoscopy | Endoscopy | Detection of Nasopharyngeal Carcinoma - pre and post treatment | Convolutional neural network | Very good |
| Li – China | 2018 | Development and validation of an endoscopic images-based deep learning model for detection with nasopharyngeal malignancies 08 Information and Computing Sciences 0801 Artificial Intelligence and Image Processing | Endoscopy | Detection of Nasopharyngeal Carcinoma | Convolutional neural network | Good |
| Girdler – South Korea | 2021 | Feasibility of a deep learning-based algorithm for automated detection and classification of nasal polyps and inverted papillomas on nasal endoscopic images. | Endoscopy | Differentiating nasal polyps and inverted papillomas | Convolutional neural network | Very good |
| Liu – USA | 2020 | Dense Depth Estimation in Monocular Endoscopy with Self-supervised Learning Methods | Endoscopy | Dense depth estimation from nasal endoscopy | Convolutional neural network | N/A |
| Soloviev – Russia | 2020 | Machine learning aided automated differential diagnostics of chronic rhinitis based on optical coherence tomography | Optic coherence tomography | Using AI to diagnose chronic rhinitis with OCT imaging | Machine learning | Excellent |
