# Supplementary material for: A Scoping Review of Artificial Intelligence Research in Rhinology
Source: Am J Rhinol Allergy. 2023 Mar 9;37(4):438–48. doi: 10.1177/19458924231162437 (PMC10273866; doi:10.1177/19458924231162437)
Supplement: sj-docx-6-ajr-10.1177_19458924231162437 - Supplemental material for A Scoping Review of Artificial Intelligence Research in Rhinology [file sj-docx-6-ajr-10.1177_19458924231162437.docx]

| **Surgical Planning and Assessment** | | | | | |
| --- | --- | --- | --- | --- | --- |
| **First author – Country of study** | **Year** | **Title** | **Aim of AI** | **Type of AI used** | **Diagnostic utility** |
| Tuan - Vietnam | 2022 | Shape Prediction of Nasal Bones by Digital 2D-Photogrammetry of the Nose Based on Convolution and Back-Propagation Neural Network. | Predicting nasal morphology based on 2D images | Convolutional neural network | Excellent |
| Khan - UK | 2021 | Automated operative workflow analysis of endoscopic pituitary surgery using machine learning: development and preclinical evaluation (IDEAL stage 0) | Automated analysis of surgical steps during endoscopic pituitary surgery | Convolutional neural network | Excellent |
| Staartjes - Switzerland | 2021 | Machine Vision for Real-Time Intraoperative Anatomic Guidance: A Proof-of-Concept Study in Endoscopic Pituitary Surgery | Identifying structures intra-operatively during endoscopic pituitary surgery | Semi-supervised convolutional neural network | N/A |
| Tong – Hong Kong | 2021 | Real-to-virtual domain transfer-based depth estimation for real-time3D annotation in transnasal surgery: a study of annotation accuracy and stability | Live intra-operative annotation of structures during endoscopic surgery | Supervised convoluted neural network | Very good |
| Wildfeuer – Germany | 2021 | Clinical Evaluation of keyword - based, computer - generated reports of sinus operations | Automatically generating surgical reports | Natural language processing | N/A |
| Bieck – Germany | 2020 | Language-based translation and prediction of surgical navigation steps for endoscopic wayfinding assistance in minimally invasive surgery. | Predicting navigational positions in endoscopy | Natural language processing, deep neural network | Bad |
| Borsting – USA | 2020 | Applied Deep Learning in Plastic Surgery: Classifying Rhinoplasty With a Mobile App | To determine whether candidates had rhinoplasty with mobile pictures | Convolutional neural network | Very good |
| Wang – China | 2021 | Hard frame detection for the automated clipping of surgical nasal endoscopic video | Automatic clipping of nasal endoscopy videos to identify hard frames | Convolutional neural network | Excellent |
